# Sequence Characterization of cDNA Sequence of Encoding of an Antimicrobial Peptide With No Disulfide Bridge from the Iranian Mesobuthus Eupeus Venomous Glands

**DOI:** 10.5812/ircmj.4024

**Published:** 2013-01-05

**Authors:** Ahmad Farajzadeh-Sheikh, Abbas Jolodar, Shamsedin Ghaemmaghami

**Affiliations:** 1 Department of Microbiology, School of Medicine, Ahvaz Jundishapur University of Medical Sciences, Ahvaz, IR Iran; 2 Department of Basic Sciences, Faculty of Veterinary Medicine, Shahid Chamran University of Ahvaz, Ahvaz, IR Iran; 3 Razi Vaccine and Serum Research Institute, Ahvaz, IR Iran

**Keywords:** Scorpions, Venoms, Antimicrobial Peptide Pharbitis, Reverse Transcriptase Polymerase Chain Reaction, Meucin-25, Mesobuthus Eupeus

## Abstract

**Background:**

Scorpion venom glands produce some antimicrobial peptides (AMP) that can rapidly kill a broad range of microbes and have additional activities that impact on the quality and effectiveness of innate responses and inflammation.

**Objectives:**

In this study, we reported the identification of a cDNA sequence encoding cysteine-free antimicrobial peptides isolated from venomous glands of this species.

**Materials and Methods:**

Total RNA was extracted from the Iranian mesobuthus eupeus venom glands, and cDNA was synthesized by using the modified oligo (dT). The cDNA was used as the template for applying Semi-nested RT- PCR technique. PCR Products were used for direct nucleotide sequencing and the results were compared with Gen Bank database.

**Results:**

A 213 BP cDNA fragment encoding the entire coding region of an antimicrobial toxin from the Iranian scorpion M. Eupeus venom glands were isolated. The full-length sequence of the coding region was 210 BP contained an open reading frame of 70 amino with a predicted molecular mass of 7970.48 Da and theoretical Pi of 9.10. The open reading frame consists of 210 BP encoding a precursor of 70 amino acid residues, including a signal peptide of 23 residues a propertied of 7 residues, and a mature peptide of 34 residues with no disulfide bridge. The peptide has detectable sequence identity to the Lesser Asian mesobuthus eupeus MeVAMP-2 (98%), MeVAMP-9 (60%) and several previously described AMPs from other scorpion venoms including mesobuthus martensii (94%) and buthus occitanus Israelis (82%).

**Conclusions:**

The secondary structure of the peptide mainly consisted of α-helical structure which was generally conserved by previously reported scorpion counterparts. The phylogenetic analysis showed that the Iranian MeAMP-like toxin was similar but not identical with that of venom antimicrobial peptides from lesser Asian scorpion mesobuthus eupeus.

## 1. Background

Venoms of the buthidae family Scorpion secreted by highly specialized gland tissues is responsible for the neurotoxic effects that recognize ion channels. They are usually rich disulfide-containing peptides (1, 2). Analysis of venom of these scorpions has shown the presence of hemolytic, and immune-modulatory functions (3). with regard to those neurotoxin peptides that were already isolated from scorpion decades ago, scorpion peptide with antimicrobial activity were shown in a number of scorpion venom (4-8). The antimicrobial peptides (AMPs) isolated from scorpions of the buthidae family are widely spread in nature and function as part of the innate defense mechanism against different kinds of pathogens (9). They exhibit direct catalytic effect on various pathogens through inserting into membrane bilayers and forming pores by “barrel-stave”, “carpet,” or “toroidalpore” mechanisms (10, 11). It is reported that positive charge peptides act together by the negative cell member of bacteria, ensuing in rapid cell death (12, 13). The AMPs which are relatively short (usually 10-50 aa) belong to a group of cationic, α-helical, amphipathic peptides with the presence of the several basic residues. Usually, according to their structure, three main groups of AMPs have been recognized, namely: linear peptides without cysteines that can adopt amphipathic α-helical structures in membrane environments; cysteine-rich peptides containing one or more disulfide bridges, forming β-sheet or both α-helix and β-sheet; linear peptides with an unusually high content of certain amino acids (e.g. Pro, His or Trp), or other special structure such as thio-ether rings (14). Analysis of components from scorpions of the buthidae family has shown the presence of linear peptides including parabutoporins from parabuthus schlechteri (15), opistoporin 1 and opistoporin 2 from opistophtalmus carinatus, hadrurin from hadrurus aztecus (16), IsCT1 and IsCT2 from opisthacanthus madagascariensis (14) and BmKn2 and BmKb1 from mesobuthus martensii (7). This group of peptides is quite different from scorpion neurotoxins which contain three or four disulfide bridges (4). They belong to the non-disulfide-bridge containing peptides (NDBPs), that exhibit bradykininpotentiating activity, antimicrobial action, hemolytic, and immune-modulatory functions (16). They can also be found in the venom of other arthropods or insects such as melittin from the venom of honeybee, mastoparan from wasps (17), lycotoxins (18) and pandinin 1 and 2 from spider pandinus imperator (19). The species M. eupeus from the Buthidae scorpion family is endemic of the Khozestan province of Iran.

## 2. Objectives

In this study we reported the identification of a cDNA sequence encoding cysteine-free antimicrobial peptides isolated from venomous glands of this species.

## 3. Materials and Methods

### 3.1. Scorpion Samples

Adult scorpions were collected in the field and transported to the Razi reference scorpion laboratory of Ahvaz. The scorpions were milked by electrical stimulation 4 days before RNA extraction, a period that allowed the toxin-producing cells of the venom glands to enter the secretory phase.

### 3.2. Total RNA Extraction and cDNA Synthesis

To prevent excessive cuticle debris and to avoid venom gland contamination, total RNA was obtained only from the first segment of the scorpion tail. Total RNA was extracted from ten homogenized venom glands of the Iranian scorpion M. eupeus using RNX plus solution (Cinna Gen, Iran) according to the manufacturer’s instructions. The purified total RNA was quantified by absorbance at 260 nm and used immediately or stored precipitated in ethanol at - 80°C until use. The RNA pellets were dissolved in water and used for cDNA synthesis immediately. First-strand synthesis was achieved with Super Script II moloney murine leukemia virus (M-MLV) RT (Fermented, Iran) in the presence of RNase inhibitor (Fermented, Iran), using the modified oligo (dT) as primer. For modifying the oligo (dT) primer, a linker containing two restriction endonucleases enzyme sites of XbaI and XholI were added to the 5´ends of the primer. The sequence of the modified oligo (dT) primer was Mod - T 5'- GGGTCTAGAGCTCGAGTCAC (T) 17. Briefly, 12 µl (2 µg each) of the extracted total RNA was incubated with 0.5 µg of the modified oligo (dT) primer at 70° C, for 10 minutes followed by a brief centrifugation. The reaction was chilled on ice for a few minutes and then added 1 µl RNasin (CinnaGen, Iran), 1 µl dNTP mixture (120 mM of each nucleotide), 2.5µl of 5 X enzyme buffer and 1 µl (200 U) of moloney murine leukemia virus (M-MulV) reverse transcriptase (CinnaGen, Iran). The reaction was incubated at 42°C for one hour followed by a brief centrifugation and then inactivation of the enzyme by heating at100°C for 10 minutes. Reverse transcriptase was omitted in the tubes corresponding to the negative controls.

### 3.3. Semi-nested RT-PCR

The amplification was followed by two-round Semi-nested PCR, using the diluted product of the first PCR as template for second PCR. The specific primers used for amplification of cDNA encoding the target genes were designed according to the sequence information from mesobuthus martensii putative antimicrobial protein b 26 which was retrieved from the NCBI Gen Bank. These primers were designed based on a conserved region close to the 5' and 3'-end of several genes for antimicrobial protein. Primer set was generated based on mRNA sequence (AY323830) for mesobuthus martensii antimicrobial protein. PCR primers: forward primer (B2-F) 5'-CGCGAATTCATGAAATCTCAGACCTTTTTCCT, corresponding to N-terminal (MKSQTFF) coding sequence of protein with a Eco RI site in the 5'-end; reverse primer (B2-R) 5'-CGCGGATTCCAGTAATTTTCCATTAGTTTTTGTAAG, corresponding to the complementary sequences of the C-terminal (LQKLMENY) coding region with a Bam HI site in the 5'-end. In order to apply the second round of PCR, primer Mod T-R (5'-CCCAGATCTCGAGCTCAGTG) was synthesized. RT-PCR reactions were carried out for cDNA template using standard reaction conditions. The reaction mixture (50 µl) contained 5 µl of the reverse transcription reaction, 0.2 µM of each primer, 250 µM of each dNTP and 1 U of Taq DNA polymerase in a standard PCR buffer. The thermo cycler was programmed as follows: Initial denaturation (95°C, 2 min), followed by 25 cycles (94°C for 40 s, annealing at 55°C for 1 min, and extension at 72°C for 1 min) and a final extension step at 72°C for 10 minutes. Then The amplification product was electrophoresed on 1% (w/v) agarose gel and visualized by staining with ethidium bromide. The PCR Products visualized in agarose gel electrophoresis were purified with a QIA quick gel extraction kit (Qia Gen, Iran) according to the manufacturer’s instructions.

### 3.4. DNA Sequencing and Sequence Analysis

Primer sets were generated using Primer 3 program (http://biotools.umassmed.edu/bioapps/primer3_www.cgi). The DNA was sequenced from both ends using a dideoxy termination method in an applied bio systems 373 DNA sequences. Each cDNA sequence was translated into the amino acid sequence using Translate tool software (http://ca.expasy.org/tools/pi_tool.html). The theoretical molecular mass for the putative mature peptides and theoretical pI value were estimated by using ProtParam pI/Mw tool program (http://www.expasy.org/tools/protparam.html). The putative signal peptide was predicted by the Signal P 3.0 program (http://www.cbs.dty.dk/sevices/SignalP). The cDNA sequences were surveyd against non-redundant public databases by using the blast x and blast n algorithms programs (http://www.ncbi.nlm.nih.gov.blast). The alignments of multiple sequences were obtained by using ClustalW1.8 program20 and edited with the BOXSHADE software (http://www.ch.embnet.org/software/BOX_form.html). The secondary structure prediction of the putative antimicrobial peptides was done by using the PSIPRED Protein Structure Prediction Software (http://bioinf.cs.ucl.ac.uk/psipred).

## 4. Results

### 4.1. Amplification and Sequencing of MeAMP-toxin Like Peptide

**Figure 1:**
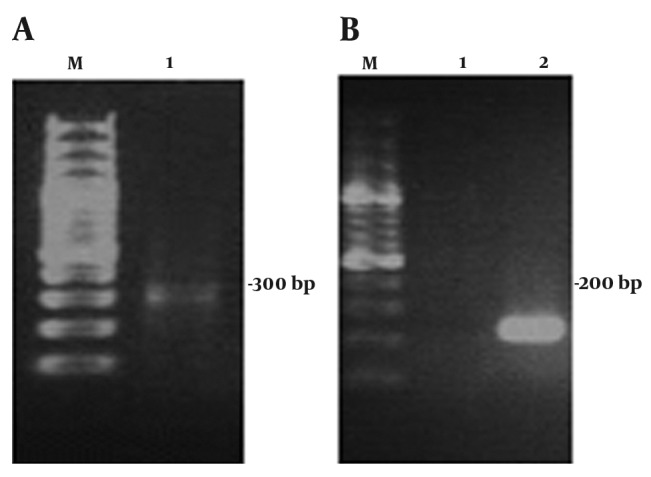
Agarose Gel Electrophoresis of RT-PCR Products from the Iranian. M. eupeus MeAMP toxin-like gene isolated from the Iranian scorpion M. eupeus A) Lane 1 is the first round of Semi-Nested RT-PCR amplification products, B) Lane 1 and 2 are negative control (water) and the second round of Semi-nested RT-PCR amplification products, respectively, Lane M in part A and B is DNA size marker. Each lane was loaded with 8 µl of the total reaction

Starting with 10 fresh venom glands giving 0.5 g of tissue, 4 µg of total RNA were obtained. After total RNA extraction, cDNA was synthesized. The full-length cDNA encoding an antibacterial peptide was amplified from the venom glands of the Iranian scorpion M. eupeus using semi-nested RT-PCR strategy. This technique allowed the amplification of the target gene in a two-round PCR method. After extracting the total RNA from M. eupeus venom glands, cDNA was synthesized by using the modified oligo dT (Mod T). The homologous primers designed to anneal to conserved sequences in the 5' and 3'-end of scorpion venom antibacterial peptide (7). For performing the first round of amplification, mod T-R primer was designed based on the modified oligo dT (mod T) primer sequence. The first round of PCR was performed by using mod T-R and B2-F primer, and the synthesized cDNA as template. The second round of PCR was performed by using B2-F and B2-R primers and one-tenth dilution of the first round of PCR as templates (Figure 1). PCR products of the second round PCR were used for nucleotide sequencing. Sequence analysis was compared with Gen Bank database using the BLAST software from NCBI Gen bank database. A 237 BP cDNA fragment encoding an antimicrobial peptide was isolated. The full-length sequence of this cDN contains an ORF encoding a peptide of 70 aa -length with a calculated molecular mass of 8.541 kDa and theoretical PI of 8.89. The calculation of monoisotopic molecular mass of the mature peptide is 1362.68 Da which is comparable with the previously peptide purified from the Brazilian scorpion Opisthacanthus cayaporum venom glands (20). The results showed that a full-length cDNA of the Iranian M. eupeus encoding the precursor of an antimicrobial peptide venom peptide with no disulfide bridge was isolated.

### 4.2. Sequence Alignment of MeAMP-toxin Like Peptide

To perform sequence comparison of the Iranian MeAMP-like toxin and other AMPs, we searched the Gen Bank database with basic local alignment search tool (BLAST) by using the entire sequence as query. We found six hits with detectable sequence similarity from venom glands to lesser Asian scorpion M. eupeus (2 hits), M. martensii (3 hits) and Buthus occitanus israelis (one hit). These hits include 6 from scorpion venom For protein. As expected the Iranian MeAMP has the highest sequence identity to the Lesser Asian M. eupeus venom antimicrobial peptide-2 and 6 (MeVAMP-2 and MeVAMP-6, 98%), It has recently been published in Database. These two precursors share high homology between each other, only being different in 3 of the 70 residues that those 3 residues existed in mature peptide. However, another venom antimicrobial peptide-9 (MeVAMP-9, 60%) from this species has less similarity with the Iranian M. eupeus. The Iranian MeVAMP cDNA shares high sequence identity (90-93%) to biologically active peptide 4, BmK1 and BmKn2, three AMPs from M. martensii, a sibling species of M. eupeus, a relatively high sequence identity (82%) to putative toxin Tx348 from buthus occitanus israelis and moderate sequence identity (60%) to MeVAMP-2 from lesser Asian mesobuthus eupeus. The lowest homology (39%) among the sequences compared was observed for cytotoxic linear peptide IsCT and IsCT2, two venom AMPs from the scorpion O. madagascariensis and pandinin-1 and pandinin-2 from emperor scorpion pandinus imperator.

### 4.3. Sequence Characterization of MeAMP-toxin Like Peptide

**Figure 2:**
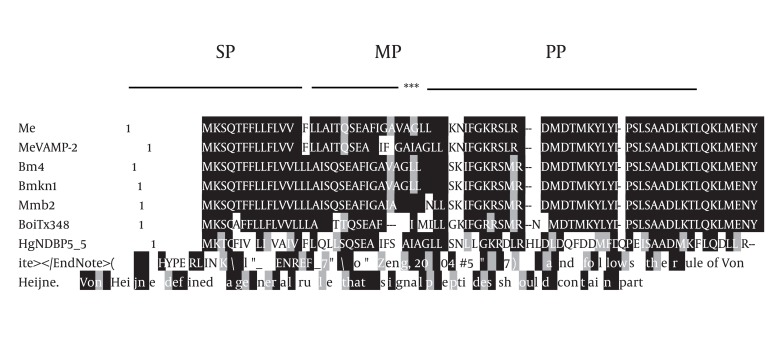
Multiple Sequence Alignment of Meamp Toxin-Like Peptide (Me) and Other Scorpion Toxins The amino acid sequence of MeAMP toxin-like peptide was aligned with venom antimicrobial peptide-2 (MeVAMP-2) (ABR20120) from species of lesser Asian scorpion M. eupeus, biologically active peptide 4 from Mesobuthus martensii (AF151795), venom peptide precursor (BmKn1) from Buthus martensii (AF150010), antimicrobial protein b2 (Mmb2) from Mesobuthus martensii, putative toxin Tx348 (BoiTx348) (FJ360839) from Buthus occitanus israelis, linear non-disulfide bridged peptide 5.5 (HgNDBP5_5) (FM998743) from Hadrurus gertschi, partial antimicrobial peptide NDPB 5.7 precursor (OcNDPB5_7) (FM998743) from Opisthacanthus cayaporum and cytotoxic linear peptide IsCT1 precoursor (OmIsCT1) (AF397895) from Opisthacanthus madagascariensis. The amino acids are denoted by one-letter symbols. Shading indicates identity (black) or conservative substitutions (grey) relative to MeAMP toxin-like peptide. Gaps represented by dashes were introduced to maximize the alignment. The cDNA encodes a signal peptide (SP), mature peptide (MP), and a propeptide (PP) were indicated. The Gly–Lye–Arg pattern that is required for posttranslational processing and the C-terminal amidation of the mature peptide is indicated by stars.

**Figure 3:**
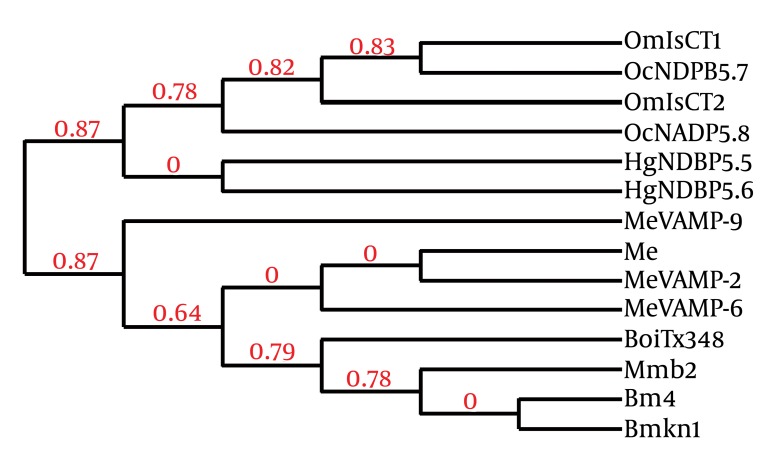
Phylogenetic Tree of MeAMP Toxin-like Peptide (Me) and Other Scorpion Counterpart Sequences Phylogeny was reconstructed based on sequences references depicted in Fig. 2, in addition to venom antimicrobial peptide-6 (MeVAMP-6) (EF445077),venom antimicrobial peptide-9 (MeVAMP-9) (EF445092) from Lesser Asian scorpion M. eupeus and also linear non-disulfide bridged peptide 5.5 (HgNDBP5_6) (P0C8W2) from Hadrurus gertschi, partial antimicrobial peptide NDPB 5.8 precursor (OcNDPB5_8) (FM998744) from Opisthacanthus cayaporum and cytotoxic linear peptide IsCT2 precoursor (OmIsCT2) (AY050522) from Opisthacanthus madagascariensis. Numbers in above the lines indicate the "branch support value" between groups.

**Figure 4:**
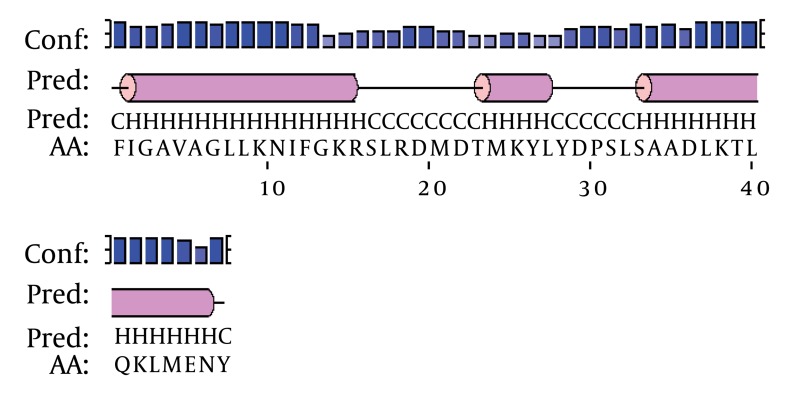
Schematic Representation for the Secondary Structure of MeAMP Toxin-like Peptide The amino acid sequences predicted to form amphipathic α-helix (H), β-sheets (E) and random coils (C) are indicated. The confidence of prediction bar (Conf) is indicated above the line.

According to von Heijne’s rule (Ser (- 3)/Ala (-1)) about cleavage sites of signal peptides (21) and signal P 3.0 prediction results, a 23-amino-acid signal peptide and the Phenylalanine at position 24were assumed to represent the start of the mature protein. We found that the overall organization of this peptide is similar to that of several scorpion antibacterial peptide precursors. In most cases, antibacterial peptides are synthesized as precursor molecules, consisting of a signal peptide that the mature peptide is located at the C-terminus of this region. Based on the results of similarity comparison (Figure 2), The precursors of MeAMP-like toxin contained a C-terminal amidation signal motif (Gly-Lys-Arg), which is processed at the C-terminal region, producing a mature peptide of 13 amino acid residues and a pro-peptide of 34 residues unusually rich in acid residues (one glu, four Asp) with unknown function. Just adjacent to the C-terminal of mature peptide, a glu was found which was removed during post-translational processing and was absolutely required for the C-terminal amidation of the mature peptide. The following two basic residues Lys–Arg were removed during carboxyl processing (22). The secondary structure prediction of MeAMP-like toxin obtained from the PSIPRED Protein Structure Prediction Software showed three α-helical conformations for residues 2 - 14, 24 - 27 and 34 - 46, separated by a random coil region including residues 16 - 23 and 28 - 33 (Figure 3). The phylogenic tree shows that the Iranian M. eupeus anitibacterial toxin-like (MeAMP-like toxin) and two toxins related to lesser Asian scorpion M. eupeus (MeVAMP-2 and MeVAMP-6) were placed, as expected, in the same group. However, in this group, MeVAMP-9 from lesser Asian scorpion M. eupeus is far more distant from from those sequences (Figure 4).

## 5. Discussion

Scorpions use a mixture of venom components designed to kill their prey or defend them against micro and macro-enemies. One group is peptides with less than 100 amino acid residues. The previous studies have reported the discovery and functional characterization of various neurotoxins with three or four disulfide bridges (4). However; little attention was paid to venom peptides with no disulfide bridge, which may be another class of valuable peptides from scorpions. These linear α-helical antimicrobial peptides with no disulfide bridge are currently described as cytolysis peptides (23). Analysis of MeAMP-toxin like peptide with those peptide counterparts let us to recognize several common features among these sequences: 1) These peptides are quite different in size and primary structure (parabutoporin with 45 residues, hadrurin with 41 residues, IsCTs and MeAMP-toxin like peptide with 13 residues with an amidated C terminus which in this case is phenylalanine, 2) They always have a strong hydrophobic feature, and a majority of them have a basic and hydrophobic residue, 3) They demonstrated amphipathic α-helical structure that is essential to lyse cell membranes (24). Because of the high degree of sequence diversification of this class of peptides, it is suggested that they might come from different ancestor in the revolution process. The signal peptide of the Iranian MeAMP had a hydrophobic segment and a positively charged amino acid residue (Lys) at the N-terminal part, which satisfies the requirements for a functional signal sequence (20). The putative signal peptide cleavage site is at a small neutral residue (Ala); moreover, the residues at position -2 and -3 are a negatively charged residue (Glu), and a small non charged residue (25), respectively (Figure 2). It is a common characteristic of signal peptides of most scorpion toxins described (7) and follows the rule of Von Heijne. Von Heijne defined a general rule that signal peptides should contain particular amino acids at position -3 and -1, which constitute the cleavage site of an endopeptidase. Residues at the position -1 should be small whereas position -3 should not contain an aromatic, charged, or large polar amino acid. The secondary-structure prediction algorithms suggest that the Iranian MeAMP is likely to consist of two distinct amphipathic α-helices joined by a flexible hinge region incorporating the prolin 53 residue (Figure 2). This arrangement appears to be a common feature and could possibly play a crucial role in their specificity against the variously charged biological membranes resulting from the association of many different phospholipids. Similar structures have been reported for OcNDPB5_7 (26) and OmIsCT1, se in those cases the hinge region is characterized by glycine residues, which are known to be helix breakers.
